# Supplementary material for: Intestinal epithelial adaptations to vertical sleeve gastrectomy defined at single-cell resolution
Source: Genomics. Author manuscript; Available in PMC 2024 Mar 22. (PMC10959023; doi:10.1016/j.ygeno.2024.110805)
Supplement: Supplemental Figures & Legends [file NIHMS1969625-supplement-Supplemental_Figures___Legends.pdf]

Supplemental Figure 1

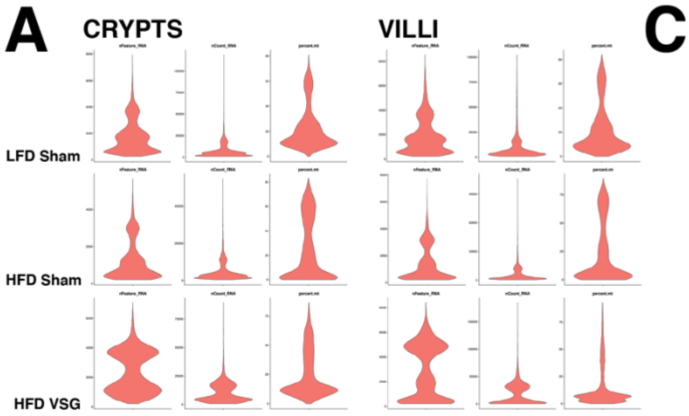

Quality Control FilteringThresholds: nFeature > 750 & percent.mt < 20%

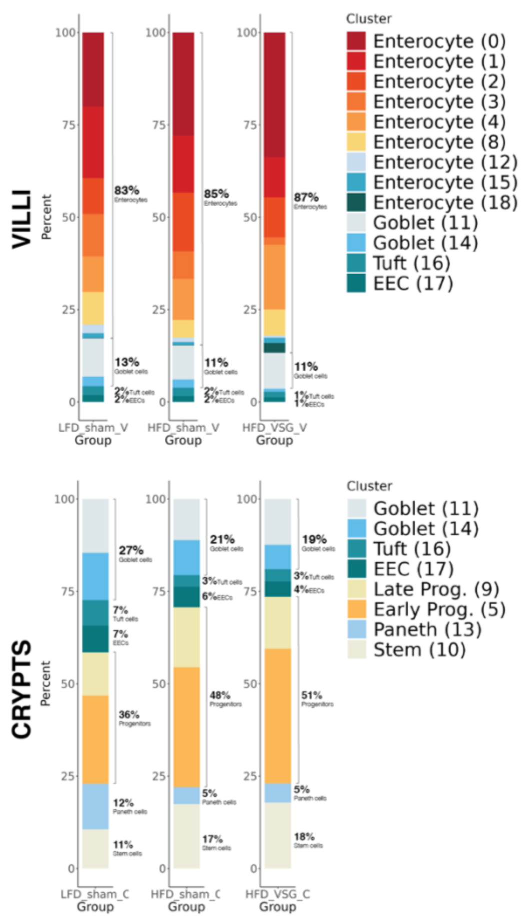

B

Sample	# Cells: Pre-Filtering	# Cells: Post-Filtering	# Cells: Immune removed
LFD Sham Crypts	9654	4168	3637
LFD Sham Villi	8733	4251	3423
HFD Sham Crypts	8374	3369	3232
HFD Sham Villi	10162	3938	3736
HFD VSG Crypts	8999	4708	4326
HFD VSG Villi	6972	4077	3490
Total	52,894	24,511	21,844

Metadata	# Cells: Pre-Filtering	# Cells: Post-Filtering	# Cells: Immune removed
LFD	18,387	8419	7060
HFD	34,507	16,092	14,784

Metadata	# Cells: Pre-Filtering	# Cells: Post-Filtering	# Cells: Immune removed
Sham	36,923	15,726	14,028
VSG	15,971	8785	7816

Metadata	# Cells: Pre-Filtering	# Cells: Post-Filtering	# Cells: Immune removed
Crypts	27,027	12,245	11,195
Villi	25,867	12,266	10,649

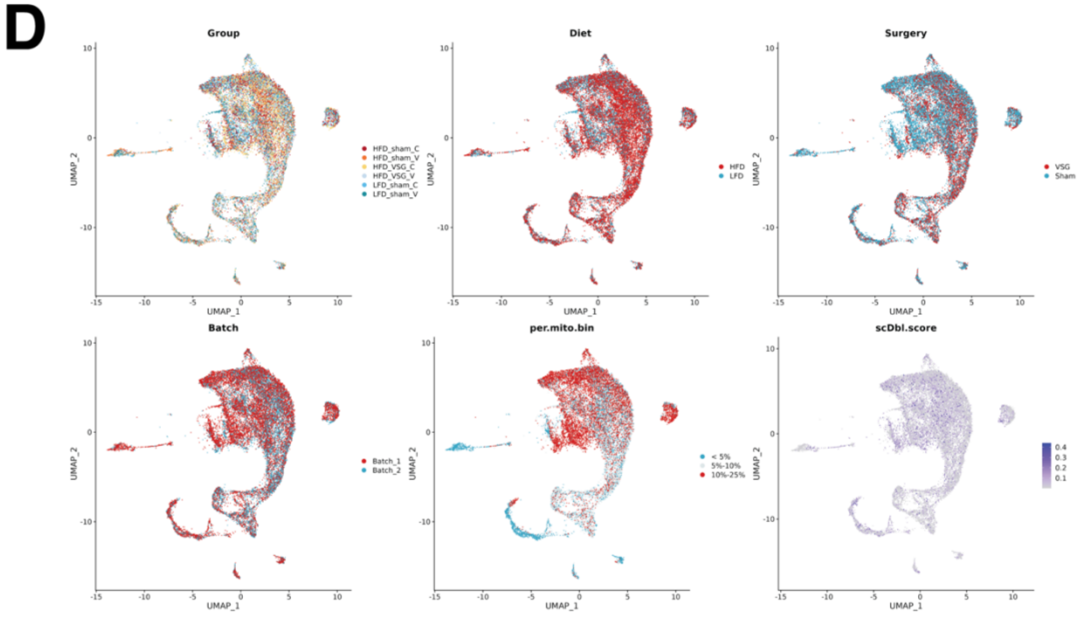

Supplementary Figure 1: Single-cell transcriptomic quality control outcomes

(A) Violin plots of the number of genes per cell (nFeature_RNA, left), the number of reads detected by unique molecular identifier (nCount_RNA, middle), and the percentage of genes per cell that map to the mitochondrial genome (percent.mt, right); each of these quality control metrics is shown for cell samples derived from the crypts (left plots) or villi (right plots) of each experimental group. (B) Tables of the number of cells from each sample group and experimental condition before and after quality control filtering; values in the rightmost column represent the finalized number of cells used in the present analyses. (C) Stacked bar plots illustrate epithelial cell composition across experimental groups as determined by the percentage of filtered cells across crypt- (bottom) and villus-based (top) clusters. (D) Uniform Manifold Approximation and Projection overlaps of various metadata, including sample group, dietary condition, surgical treatment, sample isolation batch, percentage bins of mitochondrially mapped genes, and doublet detection by scDblFinder.

Supplemental Figure 2

VILLI

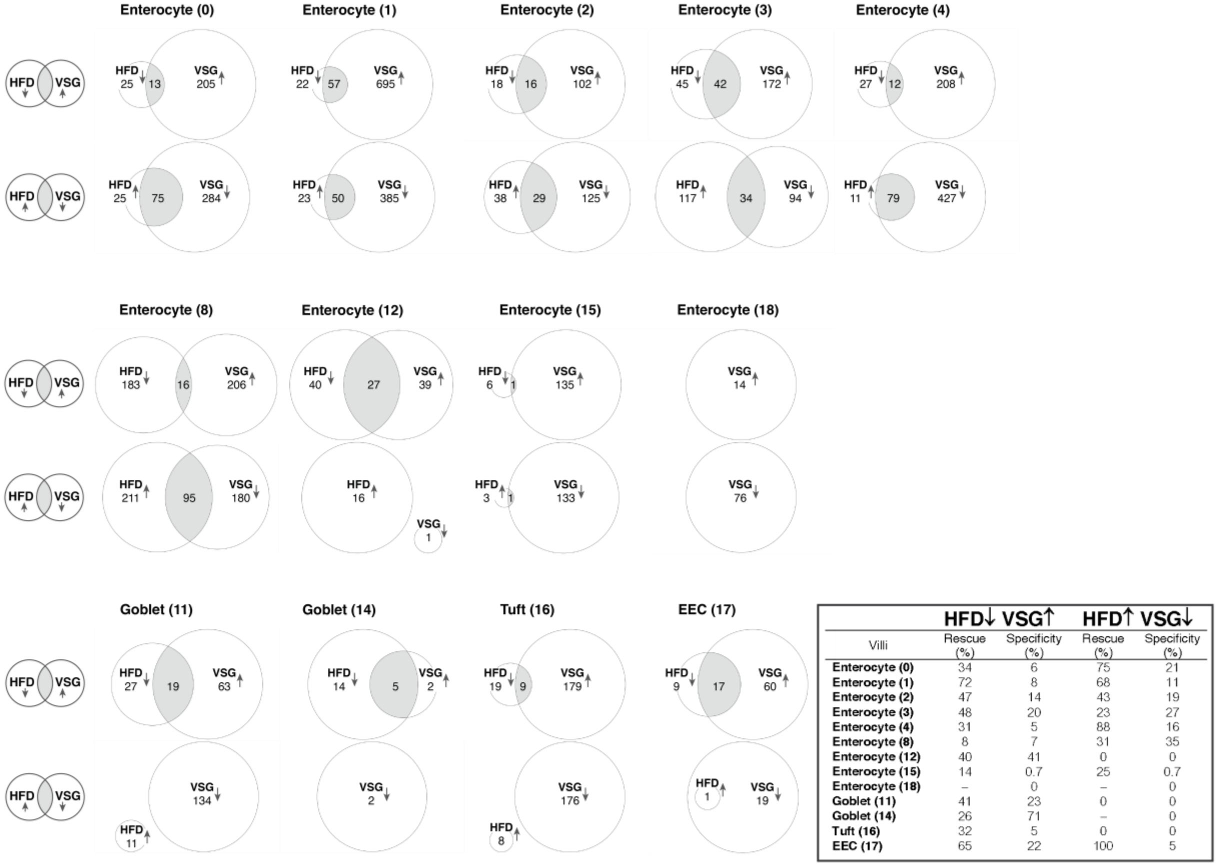

	HFD↓ VSG↑		HFD↑ VSG↓	
Villi	Rescue (%)	Specificity (%)	Rescue (%)	Specificity (%)
**Enterocyte (0)**	34	6	75	21
**Enterocyte (1)**	72	8	68	11
**Enterocyte (2)**	47	14	43	19
**Enterocyte (3)**	48	20	23	27
**Enterocyte (4)**	31	5	88	16
**Enterocyte (8)**	8	7	31	35
**Enterocyte (12)**	40	41	0	0
**Enterocyte (15)**	14	0.7	25	0.7
**Enterocyte (18)**	–	0	–	0
**Goblet (11)**	41	23	0	0
**Goblet (14)**	26	71	–	0
**Tuft (16)**	32	5	0	0
**EEC (17)**	65	22	100	5

CRYPTS

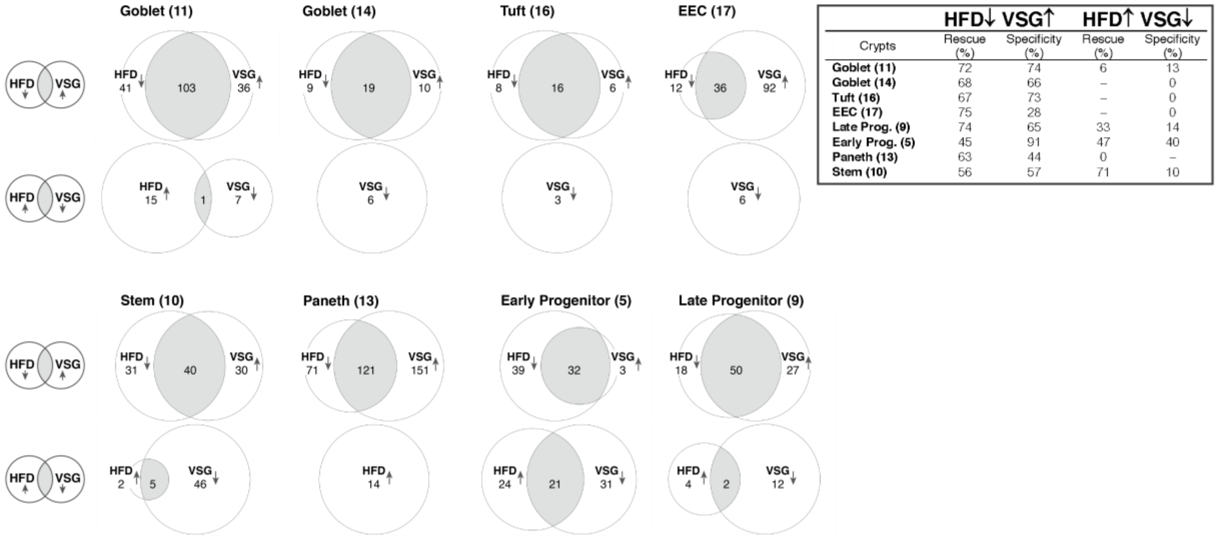

	HFD↓ VSG↑		HFD↑ VSG↓	
Crypts	Rescue (%)	Specificity (%)	Rescue (%)	Specificity (%)
**Goblet (11)**	72	74	6	13
**Goblet (14)**	68	66	–	0
**Tuft (16)**	67	73	–	0
**EEC (17)**	75	28	–	0
**Late Prog. (9)**	74	65	33	14
**Early Prog. (5)**	45	91	47	40
**Paneth (13)**	63	44	0	–
**Stem (10)**	56	57	71	10

Supplementary Figure 2: Comprehensive analysis of rescue and specificity across all intestinal epithelial cell clusters

Venn diagrams of the number of differentially expressed genes identified between dietary (HFD sham vs. LFD sham) and surgical (HFD VSG vs. HFD sham) comparisons across all crypt and villus clusters. Each cell cluster has two diagrams to illustrate both directions of differential expression (i.e., genes downregulated by HFD and upregulated by VSG, and vice versa). In some cases, there were no differentially expressed genes detected within a certain condition or shared between conditions of a given cell cluster, in which case the Venn diagram is altered accordingly. These values were used to calculate the proportions of rescue and specificity within each cell type, which are included in the accompanying tables.

Supplemental Figure 3

A

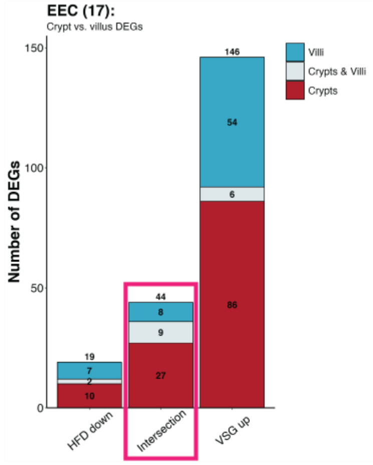

B

EEC Rescued Genes

Crypts		Crypts & Villi	Villi
Aldob	Guk1	Defa24	Defa17
Apoa1	Ndufb7	mt-Atp6	Defa21
Apoa4	Prap1	mt-Co1	Defa22
Apoc2	Prelid1	mt-Co2	Defa30
Apoc3	Rbp2	mt-Co3	Itln1
Ckmt1	Reg3b	mt-Cytb	Lyz1
Crip1	Rpl28	mt-Nd2	mt-Nd1
Dbi	Rps12	mt-Nd3	Spink4
Fabp1	Serf2	Zg16	
Fabp2	Spink1		
Fth1	Ssr4		
Gpx4	Vamp8		
Gsto1	2200002D01Rik		
Guca2b			

C

CRYPTS

Stem (10)

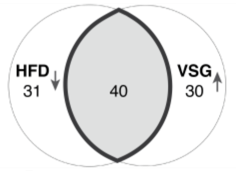

Rescue: 56%
Specificity: 57%

Paneth (13)

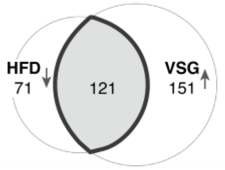

Rescue: 63%
Specificity: 44%

Rescued genes: HFD↓ & VSG↑

Stem 21	Stem & Paneth 19	Paneth 102		
2200002D01Rik	Actb	Actn4	Cox8a	mt-Nd5
Abhd2	Aldob	Anxa4	Ctnnb1	Myl6
Apoc3	Apoa1	Arpc2	Cyb5r3	Myo1a
Atp5md	Apoa4	Atp1a1	Cycs	Ndufa13
Crip1	Apoc2	Atp1b1	Dpp4	Ndufb9
Defa24	AY036118	Atp5a1	Epcam	Oaz1
Defa30	B2m	Atp5b	Espn	Pfn1
Fabp1	Cox7a2	Atp5c1	Etfb	Pgrmc1
Gpx4	Dbi	Atp5d	Ezr	Phgr1
Lct	Fabp2	Atp5e	Fau	Pigr
Mgst3	Fth1	Atp5f1	Ftl1	Prdx1
mt-Co3	Gapdh	Atp5g1	Gm42418	Reg3g
mt-Nd6	Guca2b	Atp5g3	Gsto1	Rfk
Ndufa2	Ifi27l2b	Atp5h	H2-D1	Rplp1
Plac8	mt-Atp6	Atp5j	H2-K1	Rps14
Prap1	mt-Co2	Atp5j2	H2-Q2	Rps26
Smim24	Ndufa6	Atp5l	Hadh	Scp2
Spink1	Selenop	Atp5o	Hadha	Serpinb6a
Tff3	Serf2	Calm1	Hint1	Slc25a3
Tm4sf5		Calm3	Krt8	Slc25a5
Zg16		Calml4	Lap3	Slc43a2
		Cdh17	Ldha	Slc5a1
		Cdhr2	Lgals4	Smdt1
		Cdhr5	Marc2	Tle5
		Cfl1	Mdh1	Tma7
		Chchd2	Mdh2	Tmbim6
		Cldn7	mt-Atp8	Tpm1
		Clk1	mt-Co1	Txn1
		Cox4i1	mt-Cytb	Txndc17
		Cox5a	mt-Nd1	Ubb
		Cox6b1	mt-Nd2	Uqcrb
		Cox6c	mt-Nd3	Uqcrh
		Cox7b	mt-Nd4	Uqcrq
		Cox7c	mt-Nd4l	Vil1

Supplementary Figure 3: Cell type-specific rescue of genes downregulated by HFD and upregulated by VSG

(A) Bar plot of the number of differentially expressed genes (DEGs) within the EEC cluster downregulated by HFD (left), upregulated by VSG (right), or both (middle), partitioned by location along the crypt-villus axis (color blocks). The EEC lineage shows comparable proportions of rescue and specificity between the crypt and villus compartments. (B) List of genes rescued in EECs, found in the intersection of DEGs downregulated by HFD and upregulated by VSG (corresponding with the outlined bar in panel (A). Despite similar degrees of rescue, EECs in the crypts versus villi show distinct genes rescued within each compartment. (C) List of genes rescued among the stem and Paneth clusters, as described in Figure 4C-D.
